# Revolutionizing Cardiology: The Role of Artificial Intelligence in Echocardiography

**DOI:** 10.3390/jcm14020625

**Published:** 2025-01-19

**Authors:** Bhanu Maturi, Subash Dulal, Suresh Babu Sayana, Atif Ibrahim, Manasa Ramakrishna, Viswanath Chinta, Ashwini Sharma, Harish Ravipati

**Affiliations:** 1 Department of Advanced Heart Failure and Transplantation, UTHealth Houston, Houston, TX 77030, USA; 2 Department of Medicine, Harlem Hospital, New York, NY 10037, USA; subashdulal919@gmail.com; 3 Department of Pharmacology, Government Medical College, Kothagudem 507118, India; suresh.pharmacology@gmail.com; 4 Department of Cardiology, North Mississippi Medical Center, Tulepo, MI 38801, USA; dr.atif.ibrahim@gmail.com; 5 Belfast School of Medicine, Queen’s University, Belfast BT7 1NN, UK; manasa2@outlook.com; 6 Structural Heart & Valve Center, Houston Heart, HCA Houston Healthcare Medical Center, Tilman J. Fertitta Family College of Medicine, The University of Houston, Houston, TX 77204, USA; viswanath.chinta@hcahealthcare.com; 7 Montgomery Cardiovascular Associates, Montgomery, AL 36117, USA; asharma@mcva.com; 8 Guthrie Cortland Medical Center, Cortland, NY 13045, USA; drharishravipati@gmail.com

**Keywords:** echocardiography, artificial intelligence (AI), cardiac diagnostics, ML, image analysis, cardiovascular care

## Abstract

**Background:** Artificial intelligence (AI) in echocardiography represents a transformative advancement in cardiology, addressing longstanding challenges in cardiac diagnostics. Echocardiography has traditionally been limited by operator-dependent variability and subjective interpretation, which impact diagnostic reliability. This study evaluates the role of AI, particularly machine learning (ML), in enhancing the accuracy and consistency of echocardiographic image analysis and its potential to complement clinical expertise. **Methods:** A comprehensive review of existing literature was conducted to analyze the integration of AI into echocardiography. Key AI functionalities, such as image acquisition, standard view classification, cardiac chamber segmentation, structural quantification, and functional assessment, were assessed. Comparisons with traditional imaging modalities like computed tomography (CT), nuclear imaging, and magnetic resonance imaging (MRI) were also explored. **Results:** AI algorithms demonstrated expert-level accuracy in diagnosing conditions such as cardiomyopathies while reducing operator variability and enhancing diagnostic consistency. The application of ML was particularly effective in automating image analysis and minimizing human error, addressing the limitations of subjective operator expertise. **Conclusions:** The integration of AI into echocardiography marks a pivotal shift in cardiovascular diagnostics, offering enhanced accuracy, consistency, and reliability. By addressing operator variability and improving diagnostic performance, AI has the potential to elevate patient care and herald a new era in cardiology.

## 1. Introduction

Artificial intelligence (AI), a transformative technology in medicine, enables computational programs to emulate human cognitive functions such as learning and problem solving [1]. In cardiology, AI has brought groundbreaking advancements, particularly in diagnostic, predictive, and interpretative applications. For instance, the integration of AI in analyzing data from electrocardiograms (ECGs) has transformed these simple devices into powerful predictive tools capable of identifying cardiac anomalies and forecasting future cardiovascular events [2]. Similarly, AI’s role in cardiovascular imaging has been profound, influencing every stage of the process—from image acquisition to final reporting—thereby enhancing the accuracy, efficiency, and standardization of cardiac diagnostics [3,4,5].

**Figure 1:**
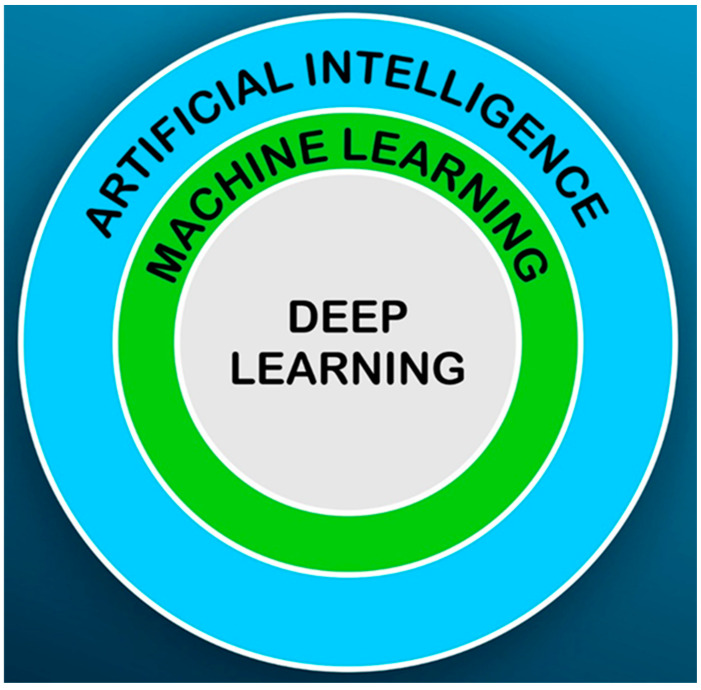
Showcasing the relationship between artificial intelligence (AI), machine learning (ML), and deep learning (DL).

**Figure 2:**
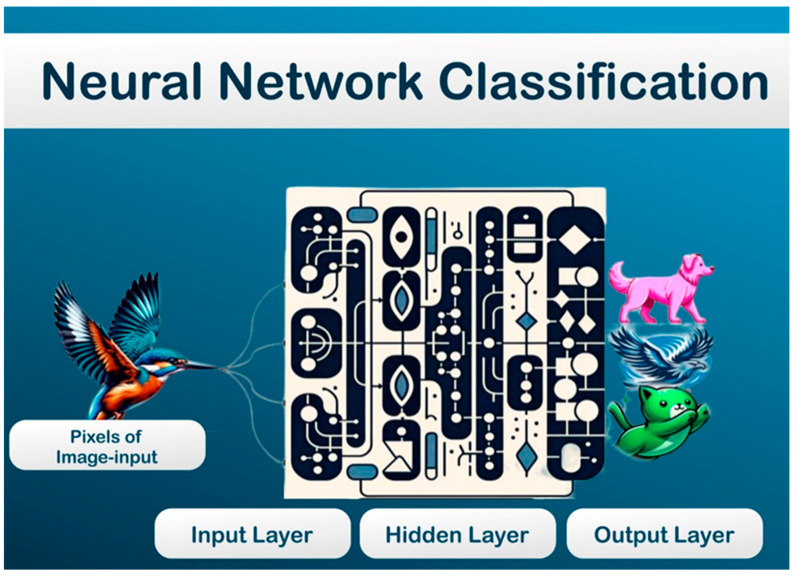
Illustration of a neural network classifying an image, with pixels as input, processed through multiple hidden layers, and resulting in classification outputs for categories like dog, bird, and cat.

One of the most impactful AI technologies in cardiology is machine learning (ML), a subset of AI that uses algorithms to learn from data and make predictions. Deep learning (DL), a specialized branch of ML, employs multi-layered neural networks to analyze complex patterns (Figure 1). These networks mimic human brain functions, enabling systems to learn and execute intricate tasks, such as automated image interpretation. DL frameworks typically consist of an input layer for data input, hidden layers for processing and weighting, and an output layer for results, making them highly effective in extracting detailed insights from medical images [6,7,8] (Figure 2). These technologies not only minimize human errors but also reduce operator variability, addressing a critical challenge in cardiac imaging.

Additionally, natural language processing (NLP), another pivotal component of AI, has emerged as a powerful tool in cardiology. NLP enables the extraction and analysis of unstructured data, such as echocardiographic reports and clinical notes, transforming it into structured information that can aid in diagnosis and reporting. By automating the interpretation of textual data, NLP can help standardize diagnostic reports, reduce documentation errors, and streamline workflows. For example, NLP algorithms can identify key findings in echocardiographic studies, flag abnormal results for further review, and generate comprehensive reports with minimal clinician input. This integration of NLP into AI-driven workflows further enhances the potential of AI to improve efficiency and consistency in cardiology practice.

Together, these AI technologies—ML, DL, and NLP—are redefining the landscape of cardiology, enabling more precise and efficient diagnostics while addressing longstanding challenges in clinical workflows.

In echocardiography, a critical diagnostic tool in cardiology, AI has demonstrated its revolutionary potential. Echocardiography is essential for real-time imaging and detection of cardiac anomalies but has traditionally been limited by operator skill and subjective image interpretation, leading to diagnostic variability and errors [6,7]. AI addresses these challenges by providing automated, consistent, and high-precision analyses of echocardiographic data. For example, AI algorithms have been applied in tasks such as automated left ventricular ejection fraction measurement, cardiac chamber segmentation, and valvular abnormality detection, all of which improve diagnostic accuracy and reduce dependence on operator expertise.

In critical care scenarios, where time and accuracy are crucial, AI’s ability to rapidly analyze echocardiographic data is particularly invaluable. This capability ensures quick and reliable diagnoses, especially for patients with hemodynamic instability [8,9,10]. Beyond critical care, AI has also been employed in predicting patient outcomes, such as identifying high-risk individuals for early intervention and optimizing treatment strategies.

AI’s integration into echocardiography and cardiology signifies a paradigm shift towards more efficient, accurate, and standardized cardiac care. Its applications encompass image acquisition, real-time interpretation, diagnosis, and prognosis evaluation, all of which contribute to elevating patient care. As AI continues to evolve, its role in enhancing echocardiographic evaluations is expected to expand, further transforming cardiac diagnostics and treatment planning [11,12].

This paper aims to provide a comprehensive examination of the burgeoning role of AI in echocardiography and its broader implications in cardiology. By exploring AI’s contributions to improving accuracy, efficiency, and standardization in echocardiographic assessments, this review highlights how these technologies are reshaping cardiac diagnostics. Additionally, the paper forecasts the future trajectory of AI in cardiac imaging, emphasizing its potential to revolutionize patient care and outcomes while maintaining a critical balance between technological innovation and the indispensable expertise of medical professionals.

## 2. AI Technologies in Echocardiography

### 2.1. Machine Learning and Deep Learning in Echocardiography

ML and DL, subsets of AI, have revolutionized echocardiography by enabling automated image analysis and interpretation. The application of ML in echocardiography can be understood through the general framework of the three primary types of ML: supervised learning, unsupervised learning, and reinforcement learning. Each of these methodologies has distinct characteristics and potential applications within the field of echocardiography, although specific examples directly from this field were not identified in the search for AI-driven tools for automated left ventricular ejection fraction measurement, segmentation of cardiac chambers, and arrhythmia detection. Below is an overview of how these ML types could theoretically contribute to echocardiography, inspired by general principles and applications in related fields.

#### 2.1.1. Supervised Learning

**Figure 3:**
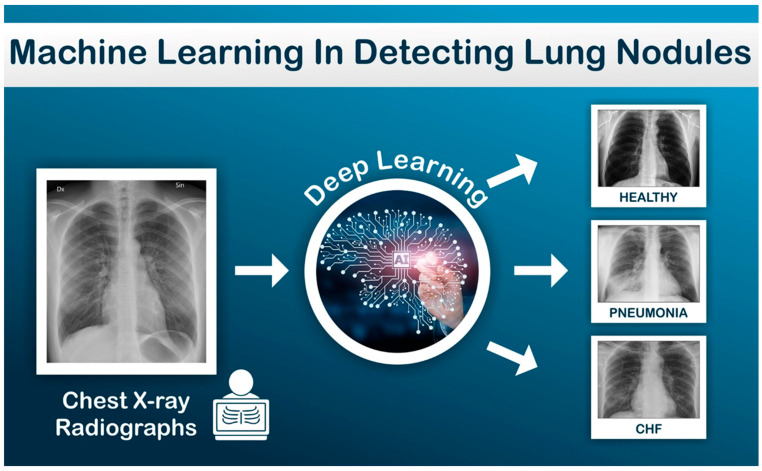
Machine learning in lung nodules utilizes advanced algorithms and data processing to transform raw data into actionable insights. By cleaning, extracting features, and training data, it powers applications like predictive analytics, natural language processing, and image recognition to classify data, predict outcomes, and detect anomalies, enhancing decision making and innovation.

Supervised learning relies on training a model using labeled input and output data to predict outcomes for new, unseen data. This approach introduces parameters that help in generating predictions. For example, supervised learning has been employed to automate the detection of lung nodules and pneumonia by analyzing chest X-rays (Figure 3). In echocardiography, this technique has proven effective for tasks such as classifying echocardiographic images into pathological categories, including hypertrophic cardiomyopathy (HCM) versus normal anatomy. Narula et al. (2016) demonstrated how supervised learning could distinguish HCM from an athlete’s heart using speckle-tracking echocardiographic data. Another notable example is the application of the EchoNet-Dynamic algorithm, which uses deep learning to calculate left ventricular ejection fraction (LVEF) with accuracy comparable to expert cardiologists. Supervised learning has also been utilized to detect valvular heart diseases, such as aortic stenosis, with high diagnostic accuracy [13]. Additionally, ML models have been employed to predict patient outcomes, such as the risk of major adverse cardiac events, based on echocardiographic and clinical data. These models streamline the interpretation of echocardiographic images, reduce inter-observer variability, and enhance diagnostic precision [4,5,6,11,13,14].

#### 2.1.2. Unsupervised Learning

Unsupervised learning algorithms identify patterns or groupings within untagged data, operating without predefined outcomes. Unlike supervised learning, this method focuses solely on recognizing hidden structures in the data. In echocardiography, unsupervised learning techniques have been used to cluster patients based on echocardiographic features to uncover novel phenotypes of cardiac diseases [15,16,17,18,19,20,21,22,23,24,25,26,27,28,29,30,31,32,33]. For example, unsupervised clustering has been employed to classify heart failure patients into subtypes based on strain patterns, leading to improved understanding of disease mechanisms and personalized treatment strategies. Loncaric et al. (2021) used unsupervised learning to categorize hypertensive patients into functional subtypes based on echocardiographic strain curves, providing valuable insights into disease progression [34]. Similarly, this method has been used to identify myocarditis in specific subgroups of systolic heart failure patients by clustering echocardiographic and clinical parameters [4,5,6]. Additionally, unsupervised learning has been applied to cluster patients with hypertrophic cardiomyopathy or dilated cardiomyopathy based on left ventricular geometry and strain data, revealing subtle differences that correlate with clinical outcomes.

#### 2.1.3. Reinforcement Learning

Reinforcement learning differs significantly from supervised and unsupervised learning, focusing on learning from the consequences of actions rather than predefined input–output pairs. Models are trained to take actions within an environment to maximize cumulative rewards. In echocardiography, reinforcement learning has been proposed for optimizing image acquisition workflows. For instance, reinforcement learning algorithms could guide sonographers in acquiring high-quality echocardiographic images by dynamically adjusting probe positioning and imaging parameters. Additionally, reinforcement learning could be applied to automate adjustments of Doppler settings to enhance blood flow visualization or optimize contrast usage during stress echocardiography. Reinforcement learning has also been explored for cardiac MRI to improve image quality in real time by adjusting scan parameters dynamically, suggesting its potential for similar applications in echocardiography [4,5,6,8].

These technologies process large datasets of echocardiographic images, learning to identify patterns and anomalies indicative of cardiac diseases. For instance, convolutional neural networks (CNNs), a type of DL, are particularly adept at image recognition tasks and have been extensively used in echocardiography to enhance diagnostic accuracy. The rapid expansion in data storage capacity and computational capabilities is significantly closing the gap in applying advanced clinical informatics discoveries to cardiovascular clinical practices. Cardiovascular imaging, in particular, offers an extensive amount of data ripe for detailed analysis, yet it demands a high level of expertise for nuanced interpretation, a skillset possessed by only a select few. DL, a branch of ML, has demonstrated considerable potential, especially in image recognition, computer vision, and video classification. Echocardiographic data, characterized by a low signal-to-noise ratio, present classification challenges. However, the application of sophisticated DL frameworks has the potential to revolutionize this field.

Narula et al. (2016) investigated the potential of ML models to improve cardiac phenotypic recognition by analyzing features of cardiac tissue deformation [13]. The study aimed to assess the diagnostic performance of an ML framework in distinguishing between HCM and physiological hypertrophy seen in athletes using speckle-tracking echocardiographic data [13]. Expert-annotated datasets were utilized to develop an automated system based on an ensemble of ML algorithms, including support vector machines, random forests, and artificial neural networks [13].

The model utilized feature selection techniques to identify critical predictors, with cardiac volume, mid-left ventricular segmental strain, and average longitudinal strain emerging as the most significant features for differentiation. Combining a majority voting approach with cross-validation ensured reliable and consistent predictions. The ML framework demonstrated superior sensitivity and specificity compared to traditional diagnostic methods, particularly in younger HCM patients and athletes with increased left ventricular wall thickness, underscoring its potential to enhance the precision of echocardiographic assessments in complex scenarios [11].

These findings highlight the potential of ML algorithms to effectively distinguish between physiological and pathological patterns of hypertrophic remodeling. This research marks progress toward developing real-time, ML-based systems for automated echocardiographic interpretation, providing valuable support for novice readers with limited experience. Such technologies aim to automate tasks traditionally performed by humans, facilitating the extraction of meaningful clinical insights from large volumes of imaging data. Moreover, the vision includes the development of contactless echocardiographic examinations, an advancement particularly relevant in the context of social distancing and public health challenges posed by the pandemic [11].

#### 2.1.4. Image Acquisition and Preprocessing with AI

**Figure 4:**
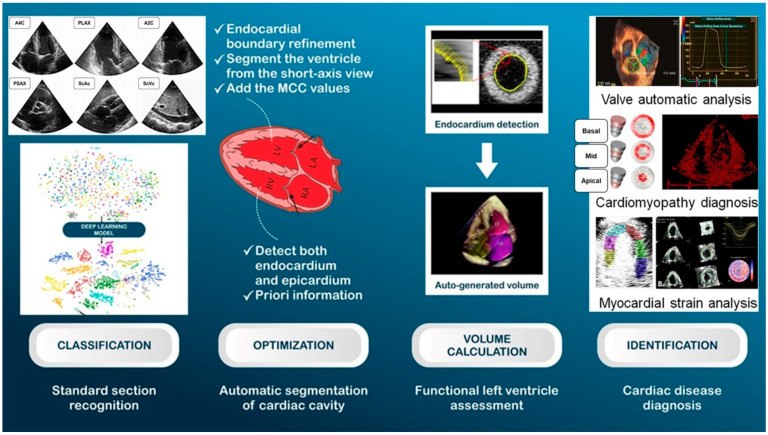
Workflow of AI in echocardiography, encompassing classification of standard sections, optimization of cardiac cavity segmentation, volume calculation for functional assessment, and identification of cardiac diseases through advanced analyses. A4C: Apical 4-Chamber View; PLAX: Parasternal Long Axis View; A2C: Apical 2-Chamber View; PSAX: Parasternal Short Axis View; ScAc: Subcostal Apical Chamber View; ScVc: Subcostal (or Subxiphoid) View—Inferior Vena Cava View.

AI significantly improves image acquisition in echocardiography. AI algorithms can assist sonographers in acquiring standard echocardiographic views, thus reducing variability and ensuring consistency in image quality. Automated border detection and image segmentation techniques also facilitate more accurate and reproducible measurements of cardiac structures and functions. A recent study explored the development of an AI system to automate stress echocardiography analysis, aiming to enhance clinician interpretation in diagnosing coronary artery disease, a leading global cause of mortality and morbidity. Employing an automated image processing pipeline, the study extracted novel geometric and kinematic features from a large, multicenter dataset, training an ML classifier to identify severe coronary artery disease. The results showed high classification accuracy, with significant improvements in inter-reader agreement and clinician confidence when AI classifications were used, underscoring the potential of AI in refining diagnostic processes in stress echocardiography [12]. Echocardiography, a widely used imaging technique in cardiovascular medicine, leverages ultrasound technology to produce high-resolution images of the heart and its surrounding structures (Figure 4). Recent advancements in deep learning, particularly the use of CNNs, have demonstrated the potential to revolutionize echocardiographic analysis. These models can identify local cardiac structures, estimate cardiac function, and predict systemic phenotypes that contribute to cardiovascular risk but are often difficult for human interpretation.

For example, the deep learning model EchoNet has been applied to accurately identify structural abnormalities such as pacemaker leads, enlarged left atrium, and left ventricular hypertrophy. It has also shown the ability to quantify cardiac parameters, including left ventricular end-systolic and diastolic volumes and ejection fraction. Furthermore, EchoNet has been utilized to predict systemic phenotypes, such as patient age, sex, weight, and height, providing valuable insights into cardiovascular health. These applications highlight the transformative role of AI in enhancing the diagnostic capabilities and clinical utility of echocardiography [12].

Semantic segmentation in 2D echocardiography is essential for evaluating cardiac functions and enhancing the diagnosis of heart diseases. However, two significant challenges remain in this field: the need for effective feature enhancement techniques to capture contextual details and the difficulty in ensuring label coherence for pixel-level category predictions. To address these issues, Liu et al. (2021) introduced the Deep Pyramid Local Attention Neural Network (PLANet), a deep learning model designed to enhance segmentation accuracy in 2D echocardiography [15]. The model incorporates a pyramid local attention module to improve feature representation by leveraging information from compact and sparse neighboring contexts [15]. Additionally, a label coherence learning mechanism was implemented to maintain consistency in pixel-level predictions by guiding the learning process with explicit supervision signals.

The PLANet model was rigorously tested on two large-scale public datasets, the Cardiac Acquisitions for Multi-Structure Ultrasound Segmentation (CAMUS) dataset and the sub-EchoNet-Dynamic dataset. Results demonstrated that PLANet outperformed both traditional and existing deep-learning-based segmentation approaches in terms of geometric and clinical metrics. Furthermore, PLANet operates in real time, making it a promising tool for supporting cardiologists in delivering accurate and efficient diagnoses. Analysis of interpretation highlights EchoNet’s ability to focus on key cardiac structures during tasks that are explainable to humans, while also identifying regions of interest when predicting systemic phenotypes that are challenging for manual assessment [15]. By applying machine learning to echocardiographic images, repetitive tasks in clinical workflows can be streamlined, preliminary interpretations can be provided in areas lacking sufficient cardiology expertise, and complex phenotypes that are difficult to evaluate manually can be predicted effectively.

#### 2.1.5. Standard View Classification

AI-driven systems can classify echocardiographic views, such as the parasternal long-axis or four-chamber views, with high accuracy. This classification is crucial for standardizing echocardiographic examinations and ensuring comprehensive cardiac evaluation. These systems use ML algorithms trained on large datasets to recognize and categorize different echocardiographic views and explore the potential of left ventricular pressure–volume (LV-PV) loops derived non-invasively from echocardiography as a comprehensive tool for classifying heart failure (HF) with greater detail. LV-PV loops are crucial for hemodynamic evaluation in both healthy and diseased cardiovascular systems. The authors employed a time-varying elastance model to establish non-invasive PV loops, extracting left ventricular volume curves from apical four-chamber views in echocardiographic videos. Subsequently, 16 cardiac structure and function parameters were automatically gathered from these PV loops. Utilizing six ML methods, we categorized four distinct HF classes and identified the most effective classifier for feature ranking. Handheld ultrasound devices have been developed to facilitate imaging in new clinical settings, but quantitative assessment has been challenging. In the field of echocardiography, quantifying the LVEF typically involves identifying endocardial boundaries manually or through automation, followed by model-based calculation of end-systolic and end-diastolic LV volumes. Recent advancements in AI have led to computer algorithms that can almost automate the detection of endocardial boundaries and measure LV volumes and function. However, boundary identification remains error-prone, limiting accuracy in certain patients. We hypothesized that a fully automated ML algorithm could bypass border detection and estimate the degree of ventricular contraction, similar to a human expert trained on tens of thousands of images.

An ML algorithm was developed to estimate LVEF automatically using a large database of echocardiographic studies, which included apical two- and four-chamber views. The algorithm, AutoEF, was tested on an independent cohort of patients, and its automated LVEF estimates were compared to reference values obtained through measurements averaged from three expert readers using a conventional volume-based technique. Agreement between the automated and reference values was assessed using statistical methods such as linear regression and Bland–Altman analysis, while the consistency of automated estimates across different apical view combinations was also evaluated.

The results demonstrated that automated LVEF estimation was feasible across all tested cases, showing high consistency and strong agreement with reference values. The sensitivity and specificity for detecting LVEF ≤35% were comparable to those obtained by clinicians using conventional methods, highlighting the algorithm’s accuracy and reliability. These findings suggest that ML-based tools like AutoEF could provide efficient and precise LVEF measurements, comparable to those obtained by expert clinicians, thus streamlining clinical workflows and enhancing diagnostic consistency [14].

A study by Aldaas et al. (2019) evaluated the use of a handheld ultrasound device for estimating LVEF compared to standard transthoracic echocardiography in patients undergoing routine echocardiographic examination [17]. The study involved both novice and experienced sonographers performing LVEF assessments. The results demonstrated a positive correlation between LVEFs obtained from the handheld device and the standard transthoracic echocardiogram. Sensitivity and specificity for detecting reduced LVEF were noted to be lower for recordings of suboptimal quality but improved significantly when the images were of adequate quality. The findings suggest that handheld ultrasound devices, when paired with appropriate software, can provide clinically useful LVEF estimates, achieving results comparable to those of experienced sonographers, even when operated by novice users [17].

In another study, Liu et al. (2023) explored the contributions of ML-derived parameters to HF classification [18]. The study utilized LV-PV loops generated from non-invasive imaging. Incorporating multiple ML-derived parameters from the LV-PV loops significantly improved the accuracy of HF classification compared to using LVEF alone [18]. The study highlighted the importance of parameters such as ventriculo-arterial coupling and ventricular efficiency in enhancing the performance of ML-based HF classification models. These findings underscore the potential of ML-driven, non-invasive approaches to improve HF diagnosis and management in clinical settings [18].

AI has been developed for echocardiography, but it has not yet undergone testing with blinding and randomization. In one study, authors conducted a blinded, randomized non-inferiority clinical trial (ClinicalTrials.gov ID: NCT0514064; no outside funding) to compare AI versus sonographer initial assessment of LVEF and evaluate the impact of AI on the interpretation workflow [19]. The primary objective of the study was to evaluate the change in LVEF between initial assessments made by AI or sonographers and the final evaluations conducted by cardiologists. The analysis focused on the proportion of cases with substantial changes in LVEF measurements. A total of 3769 echocardiographic studies were screened, with a subset excluded due to poor image quality. The results indicated that the proportion of studies with substantial changes in LVEF was lower in the AI group compared to the sonographer group, demonstrating the reliability of AI in initial LVEF assessments [19].

Furthermore, the mean absolute difference between the final cardiologist assessment and the independent previous cardiologist assessment was smaller in the AI group compared to the sonographer group. The study also highlighted that the AI-guided workflow reduced time requirements for both sonographers and cardiologists. Additionally, cardiologists were unable to distinguish between initial assessments made by AI and those made by sonographers, confirming the effectiveness of AI in this context. Overall, the findings support that initial LVEF assessments by AI are non-inferior to those performed by sonographers, offering a time-efficient and reliable alternative for quantifying cardiac function [19].

In conclusion, the ML algorithm for volume-independent LVEF estimation is highly accurate and comparable to conventional volume-based methods, offering a feasible alternative for cardiac function assessment. Recent advancements in software algorithms enable rapid, reliable LVEF measurements with minimal operator input, improving consistency and reducing time burdens on clinicians. While challenges such as data variability and integration into clinical workflows remain, these tools hold great promise for enhancing diagnostic efficiency and reproducibility in cardiovascular imaging.

#### 2.1.6. Cardiac Chamber Segmentation and Structural Quantification

Segmentation of cardiac chambers is a critical step in quantifying cardiac structure and function. AI algorithms, particularly those leveraging DL, have demonstrated the ability to accurately delineate cardiac chambers from echocardiographic images. This enables automated quantification of key parameters, such as ventricular volumes and ejection fraction, reducing the reliance on manual measurements and enhancing reproducibility. These algorithms are trained to distinguish between normal and pathological cardiac structures, providing valuable insights for both clinical decisions making and educational purposes.

A recent development involved a fully automated model for whole-heart segmentation encompassing all four cardiac chambers and great vessels using Computed Tomography Pulmonary Angiography (CTPA). Traditionally, CTPA evaluations relied on visual assessments and manual measurements, which often suffered from interobserver variability. By employing a CNN for semantic segmentation, this model generated volumetric imaging biomarkers with high accuracy and demonstrated strong correlations with invasive hemodynamic measurements. The study highlighted the model’s generalizability across different vendors and hospitals, emphasizing its potential for broad clinical application.

To integrate such AI algorithms into clinical practice, it is essential to embed these tools into existing imaging platforms and establish standardized workflows. Collaboration between AI developers and clinicians is crucial to ensure that these models are tailored to real-world needs. Additionally, providing clear interpretative outputs and user-friendly interfaces can facilitate adoption among healthcare teams. For new physicians, these algorithms offer significant educational benefits. Automated segmentation tools can serve as training aids, helping trainees understand cardiac anatomy, echocardiographic techniques, and the quantification of cardiac parameters. By reducing variability in assessments, these tools also enable new physicians to gain confidence and develop proficiency in interpreting echocardiographic data.

The use of DL-derived volumetric biomarkers in tools like CTPA further underscores the potential of AI to enhance cardiac assessments and predict hemodynamic parameters. With their ability to streamline workflows, improve diagnostic accuracy, and offer consistent results, these algorithms represent a significant advancement in cardiac imaging. However, addressing challenges such as interobserver variability and the standardization of training datasets will be key to realizing their full clinical potential [20].

#### 2.1.7. Functional Assessment Through AI

AI also aids in the functional assessment of the heart. By analyzing echocardiographic images, AI can detect subtle changes in cardiac function, which might be overlooked by human eyes. This is particularly useful in the early detection of conditions like heart failure or cardiomyopathies. AI algorithms can also predict patient outcomes based on echocardiographic parameters, offering valuable insights for patient management.

Precise evaluation of cardiac function is crucial for diagnosing cardiovascular diseases, monitoring cardiotoxicity, and guiding clinical management in critically ill patients. Traditional human assessments, although conducted by trained professionals, are limited by their reliance on a few cardiac cycles and are prone to observer variability. To address these challenges, advanced AI models like EchoNet-Dynamic have emerged, offering superior performance in tasks such as left ventricle segmentation, ejection fraction estimation, and cardiomyopathy diagnosis. Trained on echocardiogram videos, this video-based deep learning algorithm has demonstrated high accuracy and consistency, surpassing human experts in critical diagnostic tasks. It also maintains robust performance across external datasets, reinforcing its generalizability in different healthcare settings. By analyzing multiple cardiac cycles, the algorithm detects subtle variations in ejection fraction, providing a more reliable and consistent evaluation compared to traditional human analysis. This capability lays the groundwork for real-time and precise cardiovascular disease diagnosis.

To further support advancements in this field, a substantial annotated dataset of echocardiogram videos has been made publicly available, enabling continued research and development. Despite the growing integration of AI into echocardiography, its effectiveness under rigorous clinical conditions, such as blinding and randomization, has not been extensively evaluated. To address this, a randomized and blinded clinical trial was conducted to compare AI-guided LVEF assessment with traditional sonographer evaluations. The trial focused on the variance between initial assessments by AI or sonographers and final evaluations by cardiologists, particularly identifying substantial changes in measurements. The findings revealed that AI assessments resulted in fewer significant changes compared to sonographer evaluations, demonstrating improved consistency. Furthermore, the difference between the final cardiologist assessment and an independent prior assessment was smaller in the AI group, underscoring the accuracy of AI-guided evaluations.

The AI-guided approach not only reduced the time required for sonographers and cardiologists but also maintained the integrity of blinding, as cardiologists were unable to distinguish between assessments made by AI or sonographers. These findings affirm that AI’s initial assessment of LVEF is comparable to, and in some cases more consistent than, traditional methods. The study concludes that AI-based systems are a reliable and efficient alternative for echocardiographic evaluation of cardiac function, offering significant potential for integration into clinical practice [20,21].

#### 2.1.8. AI in Strain Imaging

Strain imaging in echocardiography, which assesses myocardial deformation, benefits significantly from AI. AI algorithms enhance the accuracy and reproducibility of strain measurements, important in the prognosis and management of various cardiac conditions. Recently, one study tested a novel AI method that automatically measures GLS using deep learning. It included 200 patients with varying levels of left ventricular function. The AI successfully identified standard apical views, timed cardiac events, and measured GLS with high accuracy and speed, showing potential to facilitate GLS implementation in clinical practice [22]. Another study aims to validate an AI-based tool’s performance in estimating LV-EF and LV-GLS from ECHO scans. Conducted at Hippokration General Hospital in Greece, it will involve two phases with 120 participants. The study will compare the AI tool’s accuracy and time efficiency against cardiologists of varying experience levels [23].

#### 2.1.9. Integration with Doppler Echocardiography

Doppler echocardiography, essential for evaluating blood flow and pressures within the heart, has also been augmented by AI. AI algorithms can automatically measure Doppler parameters, thus expediting the diagnostic process and enhancing precision. Yang et al. (2022) developed a cutting-edge DL framework to analyze echocardiographic videos autonomously for identifying valvular heart diseases (VHDs) [24]. Despite the advancements in DL for echocardiogram interpretation, its application in analyzing color Doppler videos for diagnosing VHDs remained unexplored. The research team meticulously crafted a three-stage DL framework that not only classifies echocardiographic views but also detects the presence of VHDs such as mitral stenosis (MS), mitral regurgitation (MR), aortic stenosis (AS), and aortic regurgitation (AR) and quantifies key severity metrics. The algorithm underwent extensive training, validation, and testing using studies from five hospitals, supplemented by a real-world dataset of 1374 consecutive echocardiograms. The results were impressive, showing high disease classification accuracy and limits of agreement (LOA) between the DL algorithm and physician estimates within acceptable ranges compared to two experienced physicians across multiple metrics. This innovative DL algorithm holds great promise in automating and streamlining the clinical workflow for echocardiographic screening of VHDs and accurately quantifying disease severity, marking a significant advancement in the integration of AI in echocardiographic diagnostics [24].

While AI technologies have shown promising results in echocardiography, challenges remain in terms of algorithm standardization, data privacy, and the need for large, diverse datasets for training. Future research should focus on addressing these challenges and exploring the integration of AI with 3D echocardiography and other advanced imaging techniques. AI technologies, particularly ML and DL, are reshaping echocardiography, enhancing image acquisition, standard view classification, structural quantification, and functional assessment. As AI continues to evolve, its integration into echocardiography promises to significantly improve diagnostic accuracy, efficiency, and patient care in cardiology.

## 3. Current Applications of AI in Echocardiography

### 3.1. Case Studies and Clinical Trials

The integration of AI in echocardiography has been extensively validated through various case studies and clinical trials, illuminating its transformative impact on cardiovascular diagnostics. Asch et al. (2019) innovatively crafted a computer vision model adept at estimating LVEF, skillfully mirroring the expertise of specialists across a vast dataset of 50,000 studies [16]. Their findings revealed a noteworthy correlation and uniformity between the automated measurements and traditional human assessments [25]. Complementing this, Reynaud et al. (2021) [25] introduced a transformative model based on the self-attention mechanism, proficient in analyzing echocardiographic videos regardless of length, pinpointing end-diastole (ED) and end-systole (ES) with precision, and accurately determining LVEF [25].

Zhang et al. (2018) [26] unveiled the inaugural fully automated multitasking echocardiogram interpretation system, engineered to streamline clinical diagnosis and treatment. This groundbreaking model successfully categorized 23 distinct views; meticulously segmented cardiac structures; evaluated LVEF; and diagnosed three specific cardiac conditions: hypertrophic cardiomyopathy, cardiac amyloid, and pulmonary arterial hypertension. Building upon this, in 2022, Tromp et al. [27] developed a comprehensive automated AI workflow. This system, adept at classifying, segmenting, and interpreting both two-dimensional and Doppler modalities, drew upon extensive international and interracial datasets, exhibiting remarkable efficacy in assessing LVEF with strong diagnostic accuracy [27].

These studies shed light on the quantification of LVEF in routine medical imaging practices. However, Point-of-Care Ultrasonography (POCUS), prevalent in emergency and critical care scenarios, takes a slightly different route. POCUS, defined as the acquisition, interpretation, and immediate clinical application of ultrasound imaging conducted bedside by clinicians instead of radiologists or cardiologists [28], facilitates direct patient–clinician interactions, thereby enhancing the accuracy of diagnoses and treatments. Its ease of portability and operation renders it invaluable for prompt diagnosis in emergency and critically ill patients [28]. Nevertheless, the effective deployment of POCUS hinges on clinicians’ proficiency in utilizing the device and interpreting the data, necessitating standardized training. To bridge this gap, the focus has shifted towards AI [28]. In 2020, the FDA greenlighted two pioneering products from Caption Health: Caption Guidance and Caption Interpretation. Caption Guidance demonstrated its capability to efficiently guide novices lacking ultrasonographic experience to capture diagnostic views after brief training [29], while Caption Interpretation aided doctors in the automatic measurement of LVEF [16]. These products were then amalgamated to forge a novel AI algorithm within the POCUS framework, enabling seamless image acquisition, quality assessment, and fully automated LVEF measurements, thereby assisting doctors in swiftly and accurately collecting and analyzing images.

Collectively, these advancements underscore the indispensable clinical value of AI algorithms. They not only elevate the precision of LVEF assessments but also simplify and streamline the diagnostic and treatment processes, significantly cutting down on time and labor costs in medical imaging

### 3.2. Comparative Analysis of AI vs. Traditional Methods

Comparative analyses have consistently shown that AI can match or surpass traditional echocardiographic methods.

Knackstedt et al. (2015) conducted a comprehensive multicenter study to evaluate the efficacy of an automated endocardial border detection method using a vendor-independent software package [30]. This software employed an ML algorithm specifically designed for image analysis. The automated technique demonstrated notable reproducibility and was found to be comparable to manual tracings in determining 2D ejection fraction, LV volumes, and global longitudinal strain [30,31]. Additionally, this correlation remained consistent when image quality was rated as good or moderate. However, a slight decrease in correlation was observed with poorer image quality. The study also revealed that the results of an automated global longitudinal strain maintained good agreement and correlation [30].

Expanding the scope beyond GLS and LV volumes, a study by Zhang et al. (2018) showcased the potential of convolutional neural networks in precisely identifying echocardiographic views and providing specific measurements such as LV mass and wall thickness [26]. In their research, a convolutional neural network model was specifically developed for the classification of echocardiographic views. Utilizing data from the segmentation model, chamber dimensions were accurately calculated in alignment with established echocardiographic guidelines [26]. This study highlights the advanced capabilities of AI in enhancing the precision and efficiency of echocardiographic analysis. The superior efficiency of AI in processing and interpreting vast datasets has been highlighted as a significant advantage over conventional echocardiographic methods, which are often time-consuming and subject to inter-operator variability.

### 3.3. Diagnostic Performance of AI Algorithms

The diagnostic capabilities of AI algorithms in echocardiography have been transformative. By integrating predictive models with high accuracy, AI has significantly advanced the classification and staging of heart failure. According to the 2021 ESC guidelines [1], heart failure is categorized into three types based on LVEF: reduced, mildly reduced, and preserved. These classifications correspond to varying degrees of systolic and diastolic dysfunction. However, there remains a need for more sensitive predictive models that can effectively stage heart failure and forecast major adverse cardiac events in patients with similar characteristics.

To address this, Tokodi et al. (2020) employed topological data analysis (TDA) to integrate echocardiographic parameters of left ventricular structure and function into a unified patient similarity network [31]. This innovative approach maps similarities across multiple echocardiographic parameters, preserving key data features and capturing the complex topological relationships inherent in high-dimensional datasets. The TDA network effectively stratified patients into distinct groups, dynamically evaluating disease progression and predicting major adverse cardiac events. These findings underscore the power of AI to uncover hidden features in echocardiographic data, enhancing early and accurate diagnosis in cardiac care.

Strain parameters derived from echocardiographic post-processing are essential for assessing myocardial deformation, offering critical insights into systolic and diastolic function. These parameters play a vital role in the early detection and prognosis of cardiac dysfunction. However, high-dimensional strain data often include redundant information, making interpretation and clinical application challenging. Traditional analysis techniques frequently lack the capacity to extract meaningful features, limiting their utility in providing comprehensive information for timely and accurate clinical decisions.

In this context, AI emerges as a revolutionary tool with advanced feature extraction capabilities. AI excels at analyzing complex, high-dimensional datasets, enabling it to uncover clinically relevant patterns and streamline the differential diagnosis of cardiac diseases. By addressing the limitations of conventional methods, AI enhances the accuracy, efficiency, and depth of cardiac diagnostics, paving the way for more precise and informed decision making in healthcare.

Narula et al. (2016) developed a composite model combining support vector machine (SVM), random forest (RF), and artificial neural network (ANN) to analyze echocardiographic images. The model effectively differentiated between physiological and pathological myocardial hypertrophy patterns, leveraging strain parameters to identify hypertrophic cardiomyopathy with high sensitivity and specificity [13].

Sengupta et al. (2016) explored the application of a cognitive computing tool designed to learn and recall multidimensional attributes of speckle-tracking echocardiography (STE) datasets. This approach emulated clinical judgment by associating patient profiles with prototypical patterns. Using an Associate Memory Classifier (AMC)-based machine learning algorithm, the study analyzed data from patients with constrictive pericarditis (CP) and restrictive cardiomyopathy (RCM) normalized against control datasets. The AMC demonstrated strong diagnostic performance in distinguishing CP from RCM, with enhanced accuracy and shorter learning curves compared to other machine learning methods, suggesting its potential for standardized and interpretable assessments, particularly for novice readers [32].

Zhang et al. (2021) employed a stacked learning strategy to integrate various classification methods using two-dimensional speckle-tracking echocardiographic and clinical parameters to develop a predictive model for coronary heart disease. This integrated model combined the strengths of multiple classifiers, achieving higher accuracy and diagnostic performance compared to individual models [33].

Researchers such as Loncaric et al. (2021) have investigated disease phenotypes based on strain curves. Using an unsupervised machine learning algorithm, their study categorized hypertensive patients into distinct functional phenotypes by analyzing strain and Doppler velocity curves across cardiac cycles [34]. Similarly, Yahav et al. (2020) developed an automated algorithm to classify strain curve patterns into physiological, non-physiological, or uncertain categories, achieving high classification accuracy [35,36].

These advancements highlight the diagnostic potential of AI in echocardiographic evaluation, establishing it as an essential tool for enhancing diagnostic accuracy and enabling standardized assessments.

### 3.4. AI in Identifying Specific Cardiac Conditions—Cardiomyopathies

AI has exhibited extraordinary proficiency in the identification of specific cardiac conditions, achieving diagnostic milestones that were previously unattainable.

Haimovich et al. (2023) demonstrated the ability of CNNs to classify cardiac diseases associated with left ventricular hypertrophy (LVH) using data from electrocardiograms (ECGs). By leveraging both 12-lead and single-lead ECGs, the model effectively differentiated between conditions such as cardiac amyloidosis, hypertrophic cardiomyopathy (HCM), hypertension, and aortic stenosis. This study highlighted the potential of deep learning to deliver accurate and reliable diagnoses across diverse cardiac conditions [36].

Similarly, AI models have shown promise in identifying specific cardiac conditions in pediatric and adult populations. For instance, a CNN-based approach has been developed to detect HCM in pediatric patients, demonstrating robust diagnostic performance across diverse subgroups. In another effort, machine learning models combining clinical, laboratory, and echocardiographic data have proven effective in predicting LVH, with techniques like SVMs achieving high diagnostic accuracy [36,37].

AI has also been applied to more complex tasks, such as distinguishing between conditions like hypertensive heart disease, HCM, and cardiac amyloidosis. For example, advanced CNN and long short-term memory (LSTM) models have outperformed traditional diagnostic methods, providing greater accuracy and consistency in detecting the underlying causes of LVH. Similarly, machine learning techniques utilizing radiomics data from cardiac imaging have successfully identified myocardial tissue alterations, offering additional insights into structural and functional abnormalities [38].

The integration of multimodal data, including ECG and echocardiographic parameters, has further enhanced the diagnostic capabilities of AI models. Multimodal fusion networks have demonstrated the ability to classify the etiology of LVH, combining the strengths of multiple data sources to improve accuracy and clinical utility. Additionally, AI has shown promise in addressing conditions like dilated cardiomyopathy (DCM), enabling patient stratification into sub-phenotypes based on distinct pathophysiological mechanisms, and improving differentiation between ischemic and non-ischemic causes of left ventricular dilation [38,39,40,41,42].

In the context of RCM and CP, AI models have proven effective in distinguishing between these conditions. Techniques such as ResNet50 and ML-based approaches utilizing echocardiographic and clinical data have achieved high diagnostic performance, streamlining clinical workflows and enabling timely intervention [43,44,45,46,47,48,49]. AI has also been employed to detect rare conditions like myocardial iron deposits in thalassemia, cardiac amyloidosis, and Takotsubo syndrome, as well as differentiating LV non-compaction from other cardiomyopathy phenotypes. These advancements demonstrate the versatility and diagnostic precision of AI across a broad spectrum of cardiac diseases [48,49,50,51,52,53,54,55].

The integration of AI into echocardiography and cardiac imaging not only enhances diagnostic accuracy but also provides prognostic insights, enabling a more preventative approach in cardiology. By leveraging AI’s capabilities to analyze and interpret complex datasets, these technologies are paving the way for more efficient and personalized cardiac care, setting the stage for future innovations in clinical practice.

## 4. Challenges and Limitations of AI Echocardiography

### 4.1. Variability in Image Quality and Analysis

AI in echocardiography has heralded a new era of diagnostic precision and patient care. However, it is not without its challenges, particularly in the realm of image quality and analysis. Dey et al. (2020) highlighted the strengths of AI in cardiovascular care, particularly its ability to improve image analysis and automate diagnostic workflows, while also identifying challenges related to variability in image quality. The study noted that AI algorithms often struggle with images that deviate from the dataset norms used during training, leading to inconsistencies in diagnostic performance across diverse clinical scenarios. This finding underscores the importance of using comprehensive and diverse training datasets to enhance AI accuracy and reliability in various settings [56].

Similarly, Madani et al. (2018) demonstrated the capability of deep learning models, particularly CNNs, to classify echocardiographic views with high accuracy. Their work highlighted the potential of AI to reduce clinicians’ workload by automating routine tasks such as view classification. However, the study also emphasized the limitations of models trained on non-diverse datasets, drawing attention to the risks of algorithmic bias and the need for balanced, representative data to ensure robust performance in real-world clinical applications [57].

These insights underscore both the challenges and opportunities of AI in echocardiography, emphasizing the critical role of diverse, high-quality training datasets to optimize clinical utility while mitigating bias and inconsistencies.

### 4.2. Ethical Considerations and Algorithm Bias

The integration of AI in echocardiography also raises significant ethical considerations. One of the primary concerns is algorithmic bias, where AI systems may exhibit biased decision making due to skewed training datasets. For instance, if an AI model is predominantly trained on data from a particular demographic, its accuracy may diminish when applied to other populations. Recent study observations were that models trained on datasets from specific populations might not perform equally well on other populations, potentially leading to diagnostic inaccuracies [58]. This study is a crucial reminder of the ethical considerations necessary in AI development and deployment, stressing the importance of varied training datasets to mitigate bias [58]. This could lead to disparities in diagnosis and treatment, exacerbating existing healthcare inequalities. Additionally, there are concerns regarding patient data security, given the sensitivity of medical data. Therefore, ensuring that AI systems in echocardiography adhere to stringent data protection standards is crucial to maintain patient trust and ethical integrity [58,59]. Furthermore, there is an ongoing debate about the accountability of AI decisions in medical settings, raising questions about responsibility in cases of misdiagnosis or treatment errors. Ensuring ethical AI deployment necessitates accountable algorithms with clear human oversight mechanisms.

### 4.3. Interoperability with Existing Healthcare Systems

Another significant challenge of AI in echocardiography is its interoperability with existing healthcare systems. For AI to be effectively integrated into clinical practice, it must seamlessly interact with various healthcare technologies, including electronic health records (EHRs), imaging software, and other diagnostic tools [60]. In this study, the authors focused on arrhythmia detection rather than echocardiography, illustrating the high potential of AI in cardiology. A significant finding was the AI’s ability to analyze vast amounts of data with high accuracy, which has implications for echocardiography, where AI can be used to interpret complex imaging data efficiently. Often, healthcare systems operate on heterogeneous platforms with varying degrees of technological advancement, which can hinder the efficient integration of AI tools. The lack of standardization across systems can also pose challenges in data exchange and interpretation, potentially leading to errors or delays in patient care. Furthermore, the successful adoption of AI in echocardiography requires significant training and adaptation by healthcare professionals. There is a need for comprehensive training programs to familiarize clinicians with AI tools and their implications for clinical practice. A recent study highlighted the importance of interoperability between AI systems and existing healthcare infrastructures, emphasizing the need for AI tools to integrate seamlessly with current clinical workflows. This integration is crucial in echocardiography, where AI must work in tandem with existing diagnostic tools and electronic health records [60,61,62,63]. Overcoming these challenges in interoperability is essential for the effective use of AI in echocardiography, ultimately enhancing clinical outcomes.

## 5. Integration of AI with Other Imaging Modalities

### 5.1. Synergies Between Echocardiography and Other Modalities

The integration of AI in medical imaging has revolutionized how we approach diagnostics, particularly in the realm of cardiovascular care. Echocardiography, when combined with other imaging modalities like CT and MRI, creates synergies that can significantly enhance diagnostic accuracy and patient outcomes. Echocardiography provides detailed functional information, while CT and MRI offer superior spatial resolution and tissue characterization. The fusion of these modalities, guided by AI algorithms, can lead to a more holistic view of cardiovascular diseases. For instance, the study by Hahn and colleagues [63] focused on assessing the severity of tricuspid regurgitation using imaging techniques. They emphasized the importance of integrating echocardiography with other imaging modalities for a more comprehensive assessment. The key finding was that echocardiography provides critical functional information, and the addition of CT/MRI could enhance anatomical understanding, which is crucial for procedural planning and decision making. This study highlights the collaboration between different imaging modalities, suggesting that a multimodal approach, potentially enhanced by AI, could lead to more accurate diagnoses and better patient outcomes in valvular heart diseases [63]. AI algorithms can integrate and analyze data from these disparate sources, offering insights that might be missed by sole human interpretation. This synergy improves the accuracy of diagnoses and assists in tailoring individualized treatment plans.

### 5.2. AI in CT and MRI

AI’s role in enhancing CT and MRI imaging is substantial. In CT, AI algorithms have shown proficiency in automating tasks such as calcium scoring, which is pivotal in assessing coronary artery disease. Previous studies explored the use of AI in automating calcium scoring in cardiac CT [64,65]. The significant finding from these studies was that AI algorithms could accurately and efficiently perform calcium scoring across CT images from various vendors. This automation represents a significant advancement in evaluating coronary artery disease, demonstrating AI’s potential to standardize and expedite diagnostic processes while maintaining high accuracy. This development is pivotal in cardiac imaging, as it could lead to quicker, more reliable assessments of cardiac risk factors [64,65]. Similarly, in cardiac MRI, AI has been instrumental in automating the quantification of left ventricular volumes and ejection fraction, traditionally time-consuming tasks [64]. Authors found that AI algorithms could accurately automate these measurements, which were traditionally time-consuming and subject to inter-observer variability. This automation streamlines the workflow and potentially improves diagnostic accuracy in cardiac MRI. Their research emphasizes the utility of AI in enhancing the efficiency and reliability of cardiac imaging analysis. These advancements not only improve efficiency but also reduce variability in interpretation. Moreover, AI-powered tools have shown promise in identifying subtle pathological features in CT and MRI images that might escape manual analysis, thereby aiding in early disease detection and intervention. The integration of AI with CT and MRI is transforming cardiac imaging into a more precise, efficient, and predictive practice, ultimately improving patient care and outcomes.

### 5.3. Multi-Modality Imaging and Comprehensive Diagnosis

The concept of multi-modality imaging, augmented by AI, is becoming increasingly vital in comprehensive cardiac diagnosis. AI’s ability to aggregate and analyze data from various imaging sources like echocardiography, CT, and MRI allows for a more comprehensive understanding of complex cardiovascular conditions. For example, in cases of cardiomyopathies, echocardiography can provide information on functional parameters, while MRI can assess myocardial fibrosis, and CT can evaluate coronary arteries. AI can integrate these data streams to offer a comprehensive diagnostic picture crucial for complex clinical decision making. Previous insights into the role of multi-modality imaging in diagnosing and managing cardiomyopathies found that combining data from echocardiography, MRI, and CT could offer a more comprehensive view of cardiomyopathies, aiding in accurate diagnosis and treatment planning. This study highlights the potential of AI in integrating and analyzing data from multiple imaging modalities, leading to a more thorough understanding of complex cardiac conditions and aiding in precision medicine [64,65,66]. This integrated approach can lead to more accurate diagnoses, better risk stratification, and optimized treatment strategies, highlighting the transformative potential of AI in cardiac imaging.

### 5.4. Clinical Decision Making and AI

AI’s integration into cardiac imaging extends beyond diagnostics to influence clinical decision making. AI models can predict disease progression, response to therapies, and even long-term patient outcomes by analyzing comprehensive multi-modal imaging data [65]. In this study, a significant finding was AI’s capability to not only enhance diagnostic processes but also predict disease progression and patient outcomes by analyzing complex imaging data. This predictive ability of AI models can revolutionize clinical decision making, enabling personalized and proactive treatment strategies. The study highlighted the shift towards a more data-driven, predictive healthcare approach facilitated by the integration of AI in cardiac imaging. These predictive insights enable clinicians to make more informed decisions, potentially leading to personalized and preemptive healthcare strategies [65]. Furthermore, AI can assist in identifying patients who would benefit most from specific interventions, thereby optimizing resource usage and improving care delivery. This aspect of AI highlights a shift from reactive to proactive healthcare, where AI-driven insights guide clinical decisions to enhance patient outcomes [66,67,68].

This comprehensive exploration into the integration of AI with various imaging modalities underlines the transformative impact AI has on enhancing cardiac imaging, diagnostics, and clinical decision making [67,68]. The synergy between different modalities, augmented by AI’s analytical prowess, is paving the way for a new era in cardiovascular care.

## 6. Role of AI in Clinical Pathways and Treatment Planning

The advent of AI in healthcare has initiated a paradigm shift in clinical pathways and treatment planning. AI’s integration into these areas is redefining the landscape of patient care, making it more data-driven, efficient, and personalized.

### 6.1. AI’s Impact on Cardiologists’ Decision-Making Processes

AI’s influence on cardiologists’ decision making is profound and multifaceted. AI algorithms are capable of analyzing vast amounts of patient data, including medical histories, diagnostic images, and genetic information, beyond the capacity of the human mind. Seetharam et al. (2019) extensively explored the implications of AI and ML in healthcare. A key finding was the capacity of AI to process and analyze complex medical data, enabling clinicians to make more informed decisions. The study emphasized that AI can identify patterns and correlations in large datasets, including patient records, which would be impossible for humans to discern quickly [69]. This capability leads to more accurate diagnoses and personalized treatment plans, significantly improving patient outcomes. This deep and nuanced analysis enables cardiologists to make more informed decisions, grounded in a comprehensive understanding of each patient’s unique clinical profile. AI tools, such as predictive analytics, can forecast potential disease progression and response to treatments, allowing cardiologists to tailor their therapeutic strategies to individual patients [70,71,72,73,74,75,76]. For instance, AI algorithms can predict adverse cardiac events, helping cardiologists to preemptively modify treatment plans to prevent such outcomes [76,77]. Baldassarre et al. (2022) reviewed the state-of-the-art applications of AI in cardiovascular imaging. They discovered that AI algorithms, particularly in imaging, could predict future cardiac events by identifying subtle patterns not easily visible to the human eye. The study also noted the potential of AI in automating routine tasks in imaging analysis, thereby reducing workloads and enhancing diagnostic accuracy. Automation and predictive capability are crucial for early intervention and personalized treatment in cardiac care [78]. This proactive approach to patient management is transforming cardiology from a reactive to a predictive discipline, enhancing the quality and precision of care.

### 6.2. Future Directions for AI in Clinical Settings

The future of AI in clinical settings is vibrant and holds immense promise. One of the key directions is the development of integrated AI systems that can seamlessly interact with EHRs, diagnostic tools, and telemedicine platforms. These integrated systems would enable the real-time analysis of patient data, facilitating immediate and informed clinical decisions. Additionally, there is an ongoing effort to develop AI models that are not only predictive but also prescriptive, suggesting optimal treatment pathways based on individual patient profiles. Another exciting development is the use of AI in personalized medicine, particularly in the field of pharmacogenomics, where AI can help determine the most effective medication based on a patient’s genetic makeup. These advancements are poised to make clinical care increasingly personalized, effective, and efficient.

#### 6.2.1. Impact on Patient Care and Outcomes

The impact of AI on patient care and outcomes is substantial and far-reaching. AI-enhanced diagnostics and predictive analytics lead to earlier detection and intervention, which is crucial in managing chronic diseases like heart failure [77,78,79,80]. AI’s ability to provide personalized treatment recommendations can significantly improve patient adherence to treatment plans, which is a critical factor in chronic disease management. Moreover, AI-powered telemedicine and remote monitoring systems enable continuous patient care outside the hospital setting, improving patient convenience and reducing hospital readmissions [79,81]. These advancements not only enhance the quality of patient care but also have the potential to reduce healthcare costs by optimizing resource utilization and preventing adverse health outcomes.

Based on these study findings, AI’s role in clinical pathways and treatment planning is pivotal, offering new dimensions to cardiologists’ decision making and significantly impacting patient care and outcomes. The integration of AI in healthcare is not just a technological upgrade but a comprehensive transformation towards a more data-driven, efficient, and patient-centric approach to medical care.

#### 6.2.2. Improvements in Diagnostic Accuracy and Efficiency

##### Patient Safety and Quality of Care

The enhancement in diagnostic accuracy and efficiency, fueled by advancements in medical technology and methodologies, has been pivotal in elevating patient safety and the overall quality of care. Modern diagnostic tools and procedures, underpinned by innovative technologies such as AI and ML, have enabled healthcare providers to detect diseases earlier and with greater precision. For instance, AI-powered imaging analysis has shown remarkable proficiency in identifying anomalies that might escape human detection, thus aiding in early intervention and potentially reducing the risk of severe disease progression [82,83,84]. Obermeyer and Emanuel’s (2016) study discussed the transformative impact of big data and ML on clinical medicine, with a particular focus on improving diagnostic accuracy. They highlighted how ML algorithms, trained on large datasets, can predict patient outcomes and disease progression with remarkable accuracy [85]. This ability to predict aids in early intervention, thus enhancing patient safety and care quality [85]. The study emphasized that leveraging big data in healthcare can lead to more informed clinical decisions, reducing the incidence of misdiagnosis and improving treatment outcomes. Enhanced diagnostic accuracy not only improves treatment outcomes but also significantly minimizes the risk of misdiagnosis, thus increasing patient safety. Additionally, the use of predictive analytics in diagnostics helps in identifying patients at high risk of certain conditions, thereby facilitating proactive management and tailored care [85]. These improvements in diagnostic processes contribute substantially to higher standards of patient care and safety.

##### Cost-Effectiveness and Accessibility of Care

The strides made in diagnostic accuracy and efficiency also have significant economic implications, contributing to the cost-effectiveness and accessibility of healthcare. Advanced diagnostic technologies, while requiring initial investments, can lead to substantial long-term savings by reducing unnecessary procedures and hospital readmissions. For example, precise diagnostics can eliminate the need for redundant testing, thus cutting down healthcare costs. A recent study demonstrated that a rapid point-of-care diagnostic platform could effectively determine disease severity and guide treatment decisions. This tool represents a significant advancement in personalized medicine, as it allows for quick and accurate assessment of patient conditions, leading to timely and appropriate treatment. This innovation not only improves the quality of care for patients with sickle cell disease but also showcases the potential for similar tools in other medical conditions [86]. Furthermore, the integration of digital technologies in diagnostics has enabled remote monitoring and telehealth services, making healthcare more accessible, especially in underserved regions [87,88]. By facilitating early and accurate disease detection, these technologies help in avoiding expensive and extensive treatments at later disease stages, thus aligning with cost-effective healthcare models. The ongoing development of portable and user-friendly diagnostic devices also plays a significant role in democratizing healthcare access, allowing patients in remote areas to benefit from advanced diagnostic tools.

### 6.3. Future Directions and Innovations

Looking forward, the trajectory of improvements in diagnostic accuracy and efficiency points towards a future brimming with innovations and transformative practices. One of the most promising areas is the continued integration of AI and ML in diagnostic procedures. Future advancements are expected to focus on refining these technologies to handle more complex and subtle medical conditions. Additionally, there is a growing trend towards personalized medicine, where diagnostics are tailored to individual genetic profiles, offering the potential for highly targeted and effective treatment strategies. Another exciting prospect is the development of smart diagnostic devices, which could provide real-time health monitoring and instant diagnostic insights, thereby revolutionizing the way healthcare is delivered. The integration of blockchain technology in managing patient data could also enhance the security and efficiency of diagnostic processes. These innovations, together with continued research and development, promise to further elevate the standards of diagnostic accuracy and efficiency, significantly impacting global healthcare practices.

The advancements, ranging from big data analytics and ML to telemedicine and blockchain technology, are crucial in enhancing patient safety and quality of care. They enable early and precise detection of diseases, tailored treatment approaches, and improved management of high-risk patients. Additionally, these technologies contribute to the cost-effectiveness and accessibility of healthcare, particularly in managing chronic diseases and in remote patient monitoring. Looking ahead, the continued evolution of AI, along with personalized medicine and secure data management, promises to further revolutionize healthcare, making it more efficient, patient-centric, and adaptable to future challenges.

## 7. Emerging Trends in AI and Cardiac Imaging

### 7.1. Potential for Personalized Medicine and Prognostic Modeling

The integration of AI in cardiac imaging is paving the way for a new era in personalized medicine and prognostic modeling. AI’s ability to analyze large datasets, recognize patterns, and learn from outcomes is revolutionizing how cardiologists approach patient care. Personalized medicine, tailored to the individual characteristics, risks, and preferences of each patient, is becoming increasingly achievable with AI-driven insights derived from cardiac imaging data.

In prognostic modeling, AI algorithms are being developed to predict patient outcomes more accurately. Johnson et al.’s (2018) study provided a comprehensive overview of the role of AI in cardiology. A key finding of this study is the potential of AI to significantly enhance diagnostic accuracy and patient management in cardiology. The authors highlighted how AI algorithms, especially deep learning models, are increasingly used to interpret complex cardiac imaging data, such as echocardiograms and MRIs. They also noted AI’s ability to integrate and analyze vast amounts of patient data, leading to more personalized and precise treatments. For example, AI could help identify patients at risk of conditions like atrial fibrillation or heart failure earlier than traditional methods. This study underscored AI’s transformative potential in cardiac care, particularly in improving diagnostics, treatment personalization, and patient outcome prediction [70]. By analyzing cardiac images alongside other patient data, AI can identify subtle signs of future cardiac events or disease progression, which might be undetectable to the human eye. One intriguing study demonstrated that ML models, trained on echocardiographic data along with patient demographics and clinical history, could predict heart failure with significant accuracy. This finding is crucial, as early prediction of heart failure can lead to timely interventions, potentially improving patient outcomes. The study also highlighted the value of ML in handling large datasets to identify patterns that may not be apparent through traditional analysis. The ability of AI to extract meaningful insights from complex echocardiographic data underlines its role in advancing cardiac diagnostics. For instance, ML models analyzing echocardiograms have been used to predict heart failure, myocardial infarction, and other cardiovascular events with a high degree of accuracy [44]. This ability to anticipate future health scenarios enables clinicians to intervene earlier, customize treatment plans, and potentially improve patient outcomes.

### 7.2. Advancing Toward Autonomous Echocardiography

A significant trend in cardiac imaging is the progression toward autonomous echocardiography, where AI and ML technologies are advancing beyond image analysis to automate the entire echocardiography process. This includes guiding image acquisition, analyzing the images, and even providing preliminary diagnoses with minimal human intervention. For example, recent research has explored the application of deep convolutional neural networks (DCNNs) for detecting regional wall motion abnormalities (RWMAs) and identifying coronary infarction territories from conventional two-dimensional echocardiographic images [89,90]. These studies demonstrate that DCNNs can achieve diagnostic performance comparable to experienced cardiologists and sonographers while significantly outperforming less experienced readers, such as residents, in identifying RWMAs and their associated territories.

Autonomous echocardiography has the potential to transform cardiac imaging by increasing access to high-quality care, particularly in underserved regions where specialized cardiologists may be scarce. It also addresses challenges related to variability and subjectivity in image interpretation, a persistent issue in cardiac imaging. By standardizing assessments and ensuring consistent accuracy, AI can enhance the reliability of echocardiographic diagnoses and contribute to more equitable and efficient healthcare delivery.

## 8. Conclusions

The advent of AI in cardiac imaging marks a transformative shift in the field, reshaping cardiac care and diagnosis. By enabling personalized medicine, enhancing prognostic modeling, and advancing towards autonomous echocardiography, AI has established itself as a cornerstone of innovation in cardiology. As these technologies mature, they are poised to improve patient outcomes, enhance healthcare efficiency, and expand access to specialized care, offering a paradigm shift toward a data-driven, precise, and patient-centered approach.

AI has become an indispensable tool in cardiology, enhancing predictive accuracy, standardizing assessments, and improving the reliability of echocardiographic evaluations. Its integration into clinical practice has enabled the detection of subtle abnormalities that might otherwise go unnoticed, contributing to more accurate diagnoses and tailored treatments. However, the role of medical professionals remains vital. Cardiologists provide essential context to AI findings, ensuring nuanced interpretation that considers patient history and clinical complexity.

The growing presence of AI in echocardiography highlights the need for continuous training and adaptation among healthcare professionals. As AI tools evolve, clinicians must stay updated on their capabilities and limitations, fostering a collaborative dynamic where human expertise complements technological advancements to optimize patient care.

Looking ahead, AI is expected to handle increasingly complex diagnostic tasks, with autonomous echocardiography on the horizon. This advancement has the potential to democratize access to high-quality cardiac care, particularly in underserved regions. Additionally, the integration of AI with wearable devices and remote monitoring systems could enable real-time cardiac monitoring, facilitating predictive analytics and early interventions.

AI’s role in personalized medicine is also a promising frontier. By analyzing individual patient data, AI can support customized treatment plans tailored to specific needs and risk profiles, moving away from a generalized approach to a more targeted and effective model.

In summary, AI is revolutionizing echocardiography by merging the precision of advanced algorithms with the essential insights of medical expertise. This synergistic relationship enhances patient care and sets the stage for a future where cardiology is increasingly precise, personalized, and accessible.

## 9. Future Prospects

Automated calculation of echocardiographic parameters, such as LVEF, has the potential to significantly streamline and accelerate assessments. The continued refinement of AI-driven algorithms will likely enable faster and more accurate parameter calculations, saving time and making echocardiography more accessible across various clinical settings, including emergency departments and remote healthcare facilities.

Future advancements may also focus on automated analysis of probe movements during echocardiographic imaging. AI systems capable of evaluating the quality and consistency of probe movements could provide real-time feedback to operators, facilitating the acquisition of high-quality images. This would be especially beneficial for less experienced practitioners, enhancing both image quality and diagnostic accuracy.

The development of real-time anatomy segmentation tools represents another exciting possibility. AI-powered systems that can segment cardiac structures during echocardiography in real time could assist practitioners in accurately identifying and assessing cardiac anatomy. Such tools would be valuable for both novice and experienced healthcare providers, improving the quality of exams and serving as a powerful training aid for medical education.

As AI technologies continue to evolve, their integration into telemedicine and remote healthcare is expected to expand. AI-enabled echocardiographic tools could allow healthcare providers to conduct assessments in underserved and remote areas, extending the reach of specialized cardiac care. Real-time analysis and transmission of data to experts for remote consultations and diagnoses would further enhance accessibility and patient outcomes.

These advancements also hold promise for medical education. AI-powered systems could be developed to provide interactive training modules simulating real echocardiographic scenarios. Such tools would offer hands-on experience and guidance, benefiting medical students, residents, and practitioners seeking to enhance their echocardiography skills. This would ultimately contribute to better training and improved competency among healthcare professionals.
